# Is There a Role of Early Neonatal Events in Susceptibility to Allergy?

**Published:** 2010-03

**Authors:** F. Gloria-Bottini, E. Bottini

**Affiliations:** *Division of Human Population Biopathology and Environmental Pathology, Department of Biopathology and Imaging Diagnostics, University of Rome “Tor Vergata”, School of Medicine, Rome, Italy*

**Keywords:** ADA_1_, bilirubin, allergy

## Abstract

**Background:**

Several studies suggest a protective role of bilirubin against oxidative damage during the neonatal period. ADA_1_*2 allele has been found associated with higher bilirubin levels in newborns and with a protective action against bronchial asthma. Thus the relation between ADA_1_ and asthma could be mediated by events occurring during the early extrauterine life. Moreover the increased prevalence of allergic diseases in western populations parallels the widespread practice of phototherapy during the neonatal period. These observations prompted us to reevaluate our previous data and show new observations.

**Methods:**

Data from 2729 previously studied subjects, from 53 subjects studied at birth and after 30 years and from a survey of phototherapy frequency in four Italian Hospital including 7392 newborns are reported.

**Results:**

ADA_1_*2 allele carriers are less represented among asthmatic subjects than in controls (*p*=0.0004). ADA_1_*2 allele carriers among newborns undergoing phototherapy for hyperbilirubinemia is higher when compared to newborns not undergoing this treatment (*p*=0.006). In infants treated by phototherapy, the maximum bilirubin level attained during the first few days of life positively correlated with the ADA_1_*2 allele dose (*p*=0.001). Among subjects studied at birth, allergic rhinitis and/or conjunctivitis are more frequent among those treated with phototherapy than among those not treated (*p*=0.046).

**Conclusions:**

These observations support our hypothesis that ADA_1_*2 allele through an increase of bilirubin level in the neonatal period protects infants from oxidative stress and favours Th_2_→Th_1_ switching thus preventing allergic manifestations in later periods of life.

## INTRODUCTION

Over the past four decades an increased prevalence of allergic diseases has been observed in most developed countries. Several causes have been proposed to explain this phenomenon. It has been suggested that decreased incidence and severity of early childhood infectious could have negatively influenced the expression of T-helper 1 (Th_1_) subpopulation of cells resulting in a prevalence of the Th_2_ subpopulation (1, 2). An important role has been attributed to the worsening environmental pollution. A decreased intake of dietary antioxidant substances could have also contributed to the rise in this class of diseases (2).

Genetic factors controlling early neonatal events could influence the switch from Th_2_ to Th_1_ subpopulations. This aspect has not received much attention.

It is well known that in the first few days of extrauterine life there is a physiologic rise of serum bilirubin due to a transitory increase of red blood cells destruction and to a deficiency of mechanisms involved in the bilirubin elimination. Since elevated levels of serum bilirubin may induce severe damages to the central nervous system, in the past four decades, phototherapy has been a widely used procedure to lower serum bilirubin levels in the newborn.

More recent studies (3, 4, 5) indicate a beneficial effect of bilirubin in the neonatal period as a result of its protective effects from secondary oxidants formed in the oxidative process although some observations suggest an interaction of other factors (6). Since the newborn is particularly susceptible to oxidative damage, bilirubin could have an important role in the regulation of neonatal events involved in the switch from Th_2_ to Th_1_ subpopulations.

Previous studies by our group have shown an association of Adenosine Deaminase locus 1 (ADA_1_) polymorphisms with allergic manifestations in children and adults (7, 8) and with bilirubin levels in the newborn (9, 10). This suggests that the effects of ADA on susceptibility to allergy could be correlated with the effects of ADA on bilirubin levels.

We present (i) an update of our previous observatios and (ii) new observations on a small sample of subjects studied at birth and after thirty years. We also show (iii) a survey of the frequency of phototherapy in several hospitals.

### Adenosine Deaminase genetic polymorphism

Adenosine deaminase (ADA) is a polymorphic enzyme present in all ammalian tissues (11). ADA is located on the long arm of chromosome 20. The ADA_1_ site controls ADA activity. Two codominant alleles, ADA_1_*1 and ADA_1_*2 are present and are associated with different enzymatic activity. The corresponding three common ADA_1_ phenotypes (12) have different degrees of enzymatic activity. ADA_1_1 is 15% more active than ADA_1_ 2/1 and 30% more active than ADA_1_ 2 (13).

The ADA tissue enzymes consist of one or more molecules of RBC ADA and one molecule of adenosine deaminase complexing protein (ADPC) (14).

ADPC is identical with CD26 (a T-cell activator molecule). ADA expression may be connected with T-cell activation (15, 16).

ADA catalyses the irreversible deamination of adenosine to inosine. In purine metabolism, the classical function of the ADA enzyme is considered to be the regulation of intra-and extracellular levels of adenosine. ADA is present on the surface of many cell types, including lymphocytes where it acts as an ectoenzyme (17). A co-stimulatory role has been observed for ADA bound with CD26 on lymphocyte surface with adenosine 1 receptors (A1R).

Current interest is focused on the effects produced by adenosine activation via surface adenosine receptors. Four adenosine receptors (A1, A2a, A2b and A3) exist on the surface of different cell types (18). In the respiratory system, the bronco-constrictor effects of adenosine are well known (19). Thus, ADA_1_ polymorphism could modulate adenosine receptor activity in the respiratory tract via the signal transduction pathway of adenosine and other mediators.

Mice studies have shown a relationship between ADA and an inflammatory phenotype in the lungs, similar to human asthma. This includes extensive mast cell degranulation, eosinophilia, activation of alveolar macrophages, increase in baseline airway resistance and AHR (20–23).

Some observations suggest that ADA activity may influence the expression of Th cytokine genes (24).

## MATERIALS AND METHODS

### Subjects

We have reconsidered the following group samples:

291 asthmatic children (sample 1) and 1417 control-children (sample 2) from the Italian population (7) (Table 1).

**Table 1:** Distribution of ADA_1_ phenotypes in asthmatic patients and in controls

	Carriers of ADA_1_*2 allele (% proportion)	Total (n°)	Chi square test of independence	ODDS Ratio

ITALIAN POPULATION				
Asthmatic children	11.4%	291	p=0.02	O.R.=0.637
Control children	16.7%	1417		95%C.I. 0.42–0.95
CHINESE POPULATION				
Asthmatic adults	10.0%	120	p=0.002	O.R.=0.318
Control adults	25.9%	116		95%C.I. 0.14–0.69

Cumulative Chi square test of independence of ADA_1_*2 allele and asthma p=0.0004.

120 asthmatic adults (sample 3) and 116 control adults (sample 4) from the Chinese Han population (8) (Table 1).

785 consecutive newborns from the Italian population (9, 10) (sample 5) (Table 2).

**Table 2:** Proportion % of carriers of the ADA_1_*2 allele among newborns treated with phototherapy for hyperbilirubinemia and among newborns not treated

	Proportion % of ADA_1_*2 allele carriers	Total (n°)

Newborns treated by phototherapy	23.6%	123
Newborns not treated by phototherapy	13.7%	662
Chi square test of independence	p=0.006	
ODDS Ratio	1.933 95%C.I. 1.741–3.178	

We performed a retrospective survey interviewing previously identified cohort studied at birth more than 30 years ago. Out of 400 subjects studied at birth only 53 were still living at the address given on clinical records. We asked these 53 if they had allergic disorders including asthma, rhinitis and conjunctivitis. All subjects with allergic manifestations reported that they had at least one positive prick test (sample 6) (Table 4).

We have carried out a survey in four Italian Hospitals in order to evaluate the proportion of newborns undergoing phototherapy in the neonatal period.

5540 newborns from Sassari (Sardinia) (sample 7), 388 from Penne (Continental Italy) (sample 8), 381 from the University of Rome La Sapienza (sample 9) and 1083 from the University of Rome Tor Vergata (sample 10) were considered (Table 5).

### Methods

ADA_1_ phenotype was determined by starch gel electrophoresis (12) in sample groups 1, 2 and 5. In sample groups 3 and 4, ADA_1_ genotype was determined by DNA analysis (8). We have shown no difference between starch gel electrophoresis and DNA analysis.

Chi square test of independence was performed by SPSS programs (25). Three way contingency table analysis was carried out according to Sokal and Rohlf (26).

## RESULTS

Table 1 shows the distribution of ADA_1_ phenotypes in asthmatic patients and in controls. In both Italian and Chinese populations a significant associations between asthma and ADA_1_ phenotype (genotype) is observed. Carriers of ADA_1_*2 allele are less represented among asthmatics versus controls suggesting a protective role of this allele against asthmatic phenotypes.

Table 2 shows the proportion of carriers of ADA_1_*2 allele among newborns treated with phototherapy for hyperbilirubinemia and among newborns not treated. The proportion of subjects carrying the ADA_1_*2 allele is significantly higher in newborn treated with phototherapy versus untreated newborns. This indicates that ADA_1_*2 allele is positively associated with higher bilirubin levels in the neonatal period.

Table 3 shows the maximum bilirubin level attained in the first few days of life in newborns treated with phototherapy. These newborns were a subsample of sample 5 in which the bilirubin level was registered in all infants during the first five days of life. A highly significant positive association is observed between a maximum bilirubin level and ADA_1_*2 allele dose.

**Table 3:** Mean values of maximum bilirubin levels (mg/dl) attained during the first few days of life in newborns treated by phototherapy in relation to ADA_1_ phenotype

	ADA_1_1	ADA_1_2−1	ADA_1_2

Mean bilirubin level (mg/dl)	11.8	13.6	15.3
S.E.	0.3	0.4	−
N°	34	11	1
Variance analysis	P=0.004		
Linear correlation	P=0.001		
Eta squared	0.23		

Table 4 shows the absolute frequencies of allergic manifestations in relation to treatment with phototherapy during the neonatal period (sample 6). Rhinitis and conjunctivitis were manifest during childhood. Among subjects with asthma, only two subjects referred to have had this manifestation during childhood. A significant positive association is observed between phototherapy and allergic rhinitis and/or conjunctivitis.

**Table 4:** Phototherapy in the neonatal period and development of allergic manifestations later in life

	Subjects not treated by phototherapy	Subjects treated by phototherapy

No allergic manifestations (a)	39	2
Asthma (b)	5	0
Rhinitis and/or conjunctivitis (c)	7	3
Test of independence	p	
a vs b vs c	0.034	
a vs b	N.S.	
a vs c	0.046	

Table 5 shows the proportion of newborns treated by phototherapy in relation to locality and gender. There is a strong heterogeneity among Hospitals reflecting the different criteria adopted to treat the newborn with phototherapy. The proportion of newborns treated reached a maximum in males from Penne (33.7%) and a minimum in females from Rome Tor Vergata (4.9%). In general the frequency of phototherapy was lower in females.

**Table 5:** Proportion (percent) of newborns treated with phototherapy in relation to locality and gender

	Locality							
	Sassari		Penne		Roma La Sapienza		Roma Tor Vergata	
	Males	Females	Males	Females	Males	Females	Males	Females

Proportion (%) of newborns treated by phototherapy	22.1%	18.7%	33.7%	24.5%	12.0%	13.1%	7.6%	4.9%
N°	2860	2680	184	204	175	206	576	507
Three way contingency table analysis by a log-linear model (x=sex; y=phototherapy; z=locality)								
Interaction xyz	p=N.S.							
Association xy	p<0.005							
Association yz	p<<0.001							

## DISCUSSION

The data shown in Tables 1 and 2 show a negative correlation between ADA_1_*2 allele and asthma and a positive correlation between ADA_1_*2 allele and bilirubin levels in the neonatal period. Parallel to these correlations are two epidemiological data: the widespread practice of phototherapy in the neonatal period and the rise of asthma and other allergic manifestations in Western populations. Preliminary data reported in Table 4 suggest a positive relationship between phototherapy and allergy.

Because of the protective role of bilirubin from oxidative damage during the neonatal period, we speculate that the relationship between ADA_1_*2 allele and asthma is mediated in part by the relationship between ADA_1_*2 and bilirubin level in the newborn. Our data support the hypothesis that ADA_1_*2 favouring high level of bilirubin has a beneficial effect on oxidative damage and in turn protects from allergic manifestations later in life.

Substances that contribute to antioxidant defence protect from asthma (2): it is possible that oxidative damage of respiratory tract in the neonatal period may represent a predisposing factor to asthmatic manifestations later in life.

The role of oxidative stress on the switch Th_2_→Th_1_ subpopulations during the neonatal period has not been fully studied. Increasing bilirubin level ADA_1_*2 allele could decrease oxidative stress favouring Th_2_→Th_1_ switch thus explaining the negative correlation between ADA_1_*2 and asthma.

The positive relationship between phototherapy and allergy observed in the small sample of subjects studied at birth and after 30 years supports our hypothesis. The decrease of bilirubin level induced by phototherapy favours the oxidative stress resulting in an increased susceptibility to allergy. However, the possibility of a direct negative effect of light on oxidative stress and on Th_2_→Th_1_ switch cannot be excluded.

The long term iatrogenic consequences of phototherapy in the population are not yet known. An epidemiological study on a larger sample of subjects examined at birth and after few decades is needed.
